# Insulin-like growth factor-1 endues monocytes with immune suppressive ability to inhibit inflammation in the intestine

**DOI:** 10.1038/srep07735

**Published:** 2015-01-15

**Authors:** Rong-Ti Ge, Li-Hua Mo, Ruijin Wu, Jiang-Qi Liu, Huan-Ping Zhang, Zhigang Liu, Zhanju Liu, Ping-Chang Yang

**Affiliations:** 1 Department of Gastroenterology, The Shanghai Tenth People's Hospital of Tongji University, Shanghai, 200072, China; 2 Shenzhen Key Laboratory of Allergy & Immunology, Shenzhen University School of Medicine and State Key Laboratory of Respiratory Disease for Allergy at Shenzhen University, Shenzhen, 518060, China; 3 Brain Body Institute, McMaster University, Hamilton, ON, Canada L8N 4A6

## Abstract

The pathogenesis of some chronic inflammation such as inflammatory bowel disease is unclear. Insulin-like growth factor-1 (IGF1) has active immune regulatory capability. This study aims to investigate into the mechanism by which IGF1 modulates the monocyte (Mo) properties to inhibit immune inflammation in the intestine. In this study, the production of IGF1 by intestinal epithelial cells was evaluated by real time RT-PCR and Western blotting. Mos were analyzed by flow cytometry. A mouse colitis model was created with trinitrobenzene sulfonic acid. The results showed that mouse IECs produced IGF1, which could be up regulated by exposure to CpG-ODN (CpG-oligodeoxynueleotides) in the culture. Culture the CpG-ODN-primed IEC cells and Mos or exposure of Mos to IGF1 in the culture induced the Mos to express IL-10. The IGF1-primed Mos showed the immune suppressive effect on inhibiting the immune inflammation in the mouse colon. In conclusion, the IGF1-primed Mos are capable of suppressing immune inflammation in the intestine.

The pathogenesis of inflammatory bowel disease (IBD) is not fully understood. It is accepted that the nature of IBD is an inflammatory disorder^1^, in which the abnormality of immune response in the local tissue plays a critical role^2^. The prevalence of IBD has reached 0.1–0.5% in the world, and continued rising despite of the research about IBD has been advancing rapidly in this area in the recent years^3^. The therapeutics of IBD is unsatisfactory currently^4^. Therefore, it is urgent to bring forth new ideas to innovate novel remedies for the treatment of IBD.

Monocytes (Mos), one of the major subtypes of the white blood cells, constitute about 2%–10% of the whole leukocytes. The signature marker of Mos is CD14 or CD14 and CD16^5^. Under given micro environment, Mos differentiate into dendritic cells or macrophages, which are directly involved in multiple immune responses^6^. After activation, Mos may differentiate into several cell types, including dendritic cells, macrophages and myeloid derived suppressor cells (MDSC)^7^. The immune suppressive effect of MDSC is well recognized in tumor studies, in which MDSCs are highly immunosuppressive on effector immune cell activities^8^. To date, the underlying mechanism by which the naïve Mos differentiate into suppressive Mos has not been fully defined.

Intestinal epithelial cells (IEC) are critical components of the intestinal epithelial barrier. IECs also communicate between the external environment (the intestinal tract) and the internal environment (intestinal mucosa)^9^. The external stimulation may activate IECs and induce IECs to produce molecules to influence the immune cell functions in the sub-epithelial region^10^. IECs express a number of Toll like receptors (TLR) and can respond to the stimuli of microbes in the intestinal tract^11^. IECs produce a number of molecules, including the transforming growth factor (TGF)-β^12^. Whether IECs produce some other growth factors, such as insulin-like growth factor-1 (IGF1), has not been fully understood.

IGF1, also called somatomedin C, is a member of the IGF family. IGF is mainly produced by the liver that is regulated by the growth hormone^13^. IGF1 is one of the most potent natural activators of the AKT signaling pathway, a stimulator of cell growth and proliferation of multiple cell types in the body^14^. The IGF system has been implicated in the oncogenesis of essentially all solid and hematologic malignancies^15^. This has led to a search for specific inhibitors of the IGF receptor for cancer therapy^16^. Clinical studies have revealed a correlation between IGF and inflammation in the intestine^17^,^18^. Whether IGF can regulate the inflammatory process in the intestine has not been investigated.

IECs express several Toll like receptors (TLR), including TLR2, TLR3, TLR4, TLR9, etc^19^. Upon exposure to ligands of the TLRs, IECs can be activated. It is reported that TLR9 can recognize CpG-ODN (oligodeoxynueleotides) to activate IECs^20^. Our recent work reveals that IECs produce IGF1^21^. It has not been investigated whether CpG-ODN induces IECs to produce IGF1. Thus, we carried out the present study. The results showed that intestinal epithelial cells expressed IGF1; the latter promoted the expression of IL-10 in Mos. The IL-10-producing Mos inhibited the experimental colitis in mice.

## Results

### Intestinal epithelial cells produce IGF1

**Figure 1:**
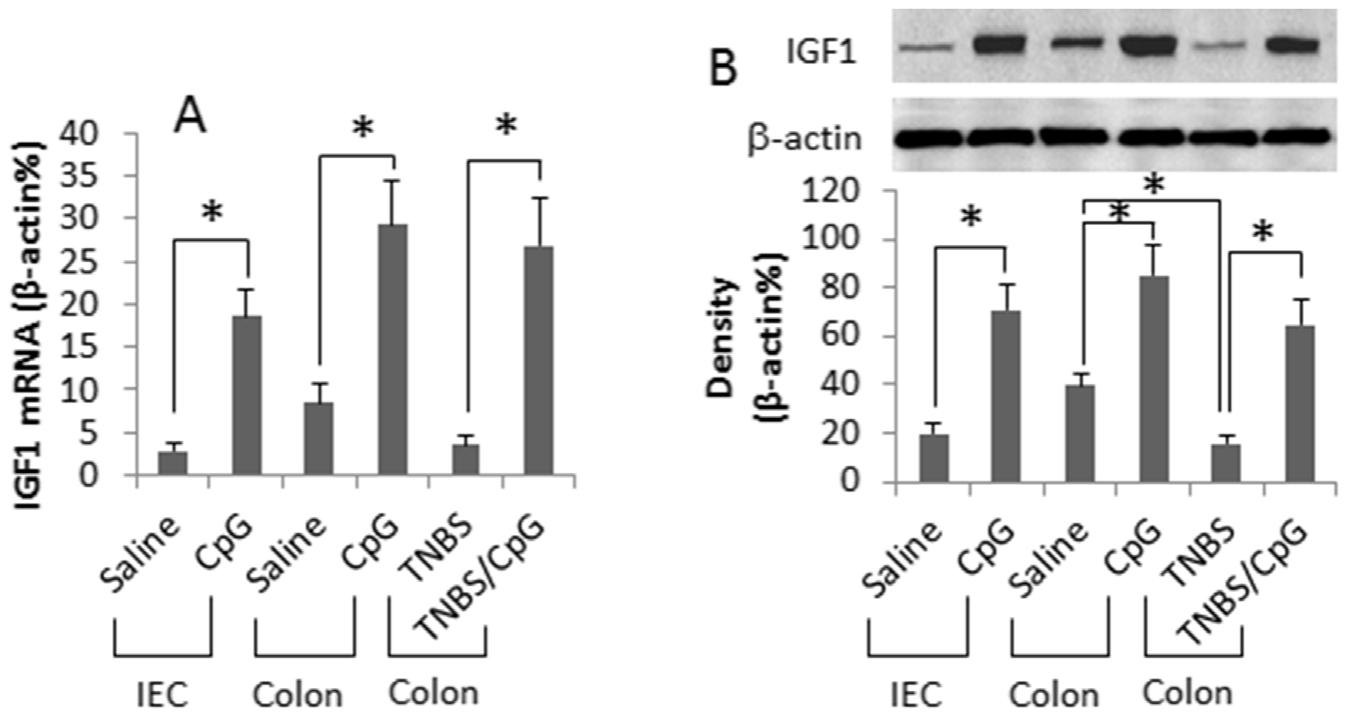
Intestinal epithelial cells express IGF1. Mouse intestinal epithelial cells (IEC, a cell line) were cultured in the presence of CpG-ODN (CpG, in short; 500 ng/ml) for 48 h. Naïve BALB/c mice or colitic mice (6 mice/group) were gavage-fed with saline or CpG (10 μg/mouse in 0.3 ml saline) daily for 6 days. The epithelia were scrapped from the colon of the mice. The samples were analyzed by RT-qPCR and Western blotting. (A) the bars indicate the mRNA levels of IGF1. (B) the Western blots indicate the IGF1 protein contents. The bars below the blots indicate the integrated density of the blots. TNBS: Colitic mice induced by TNBS. The data of bars are presented as mean ± SD. *, p < 0.01, compared with saline group. #, p < 0.01, compared with group ″0″. The data are representatives of 6 independent experiments.

Published data indicate that both IGF1^22^ and CpG-ODN^23^ are involved in regulating in the intestinal epithelial cell (IEC) activities. Whether CpG-ODN regulates IGF1 expression in IECs has not been investigated. To this end, IECs were treated with CpG-ODN in the culture. As analyzed by RT-qPCR and Western blotting, the naïve IECs expressed low levels of IGF1, which was markedly increased by exposing IECs to CpG-ODN in the culture. To enforce the results, we measured the expression of IGF1 in mouse intestinal epithelia. The results showed a low level of IGF1 in the intestinal epithelia of naïve mice, which was markedly increased by gavage-feeding with CpG-ODN daily for 6 days. In addition, we collected colonic epithelial specimens from colitic mice, in which the IGF1 levels were significantly lower than the mice treated with saline. After treatment with CpG-ODN for one week, the IGF1 levels were enhanced markedly (Fig. 1). The results indicate that intestinal epithelial cells express IGF1 that can be up regulated by CpG-ODN.

### Epithelial cell-derived IGF1 modulates Mos phenotypes in the intestine

**Figure 2:**
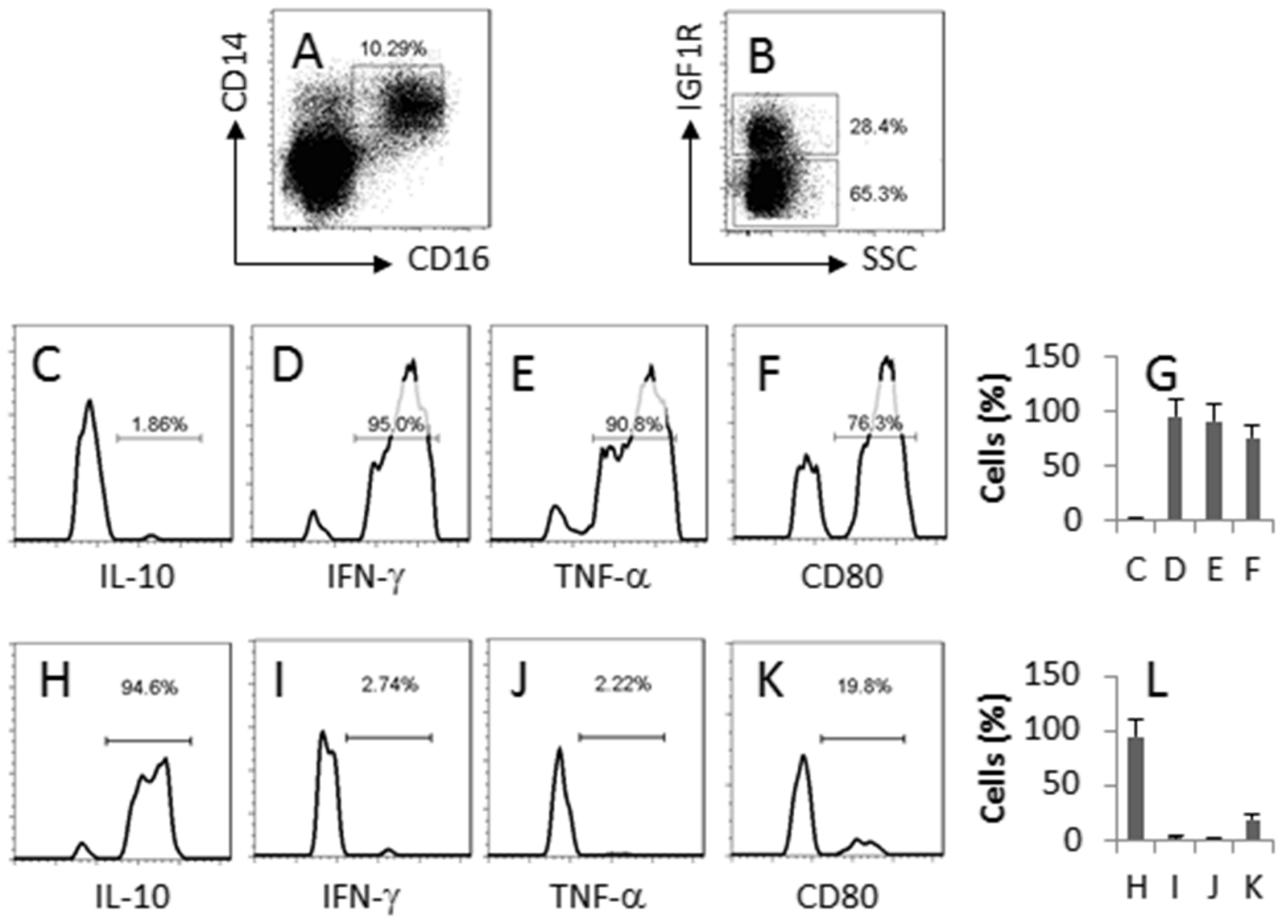
Intestinal Mos express IGF1R. Lamina propria mononuclear cells (LPMC) were isolated from the naïve BALB/c mouse colon, stained with the indicated antibodies (denoted in each sub-panel) and analyzed by flow cytometry. (A) the dot plots indicate the frequency of Mos in LPMC. (B) the gated cells of panel A were further analyzed; the cells of upper gate are IGF1 receptor positive Mos; the cells of the lower gate are IGF1 receptor negative Mos. (C–F) the histograms indicate the phenotypes of the IGF1 receptor positive Mos; the cytokine profile is denoted in each histogram. (G) the bars indicate the summarized data of C–F. (H–K) the histograms indicate the phenotypes of the IGF1 receptor negtative Mos; the cytokine profile is denoted in each histogram. (L) the bars indicate the summarized data of H–K. The data are a representative of 3 independent experiments.

Mos are one of the major subtypes of white blood cells, and can extravasate to the peripheral tissue, including the intestine. Thus, the IEC-derived IGF1 may regulate Mo activities in the intestine. To test the inference, we isolated Mos from the mouse intestine. The CD14^+^ F4/80^-^ Mos were gated and analyzed by flow cytometry (Fig. 2A). The results showed that about 28.4% Mos from the naïve mouse colon expressed IGF1R (Fig. 2B). Further analysis showed that the IGF1 receptor positive Mos were also IL-10^+^ IFN-γ^-^ TNF-α^-^ CD80^low^ (Fig. 2C–G) while the IGF1 receptor negative Mos were IL-10^-^ IFN-γ^+^ TNF-α^+^ CD80^high^ (Fig. 2H–L).

**Figure 3:**
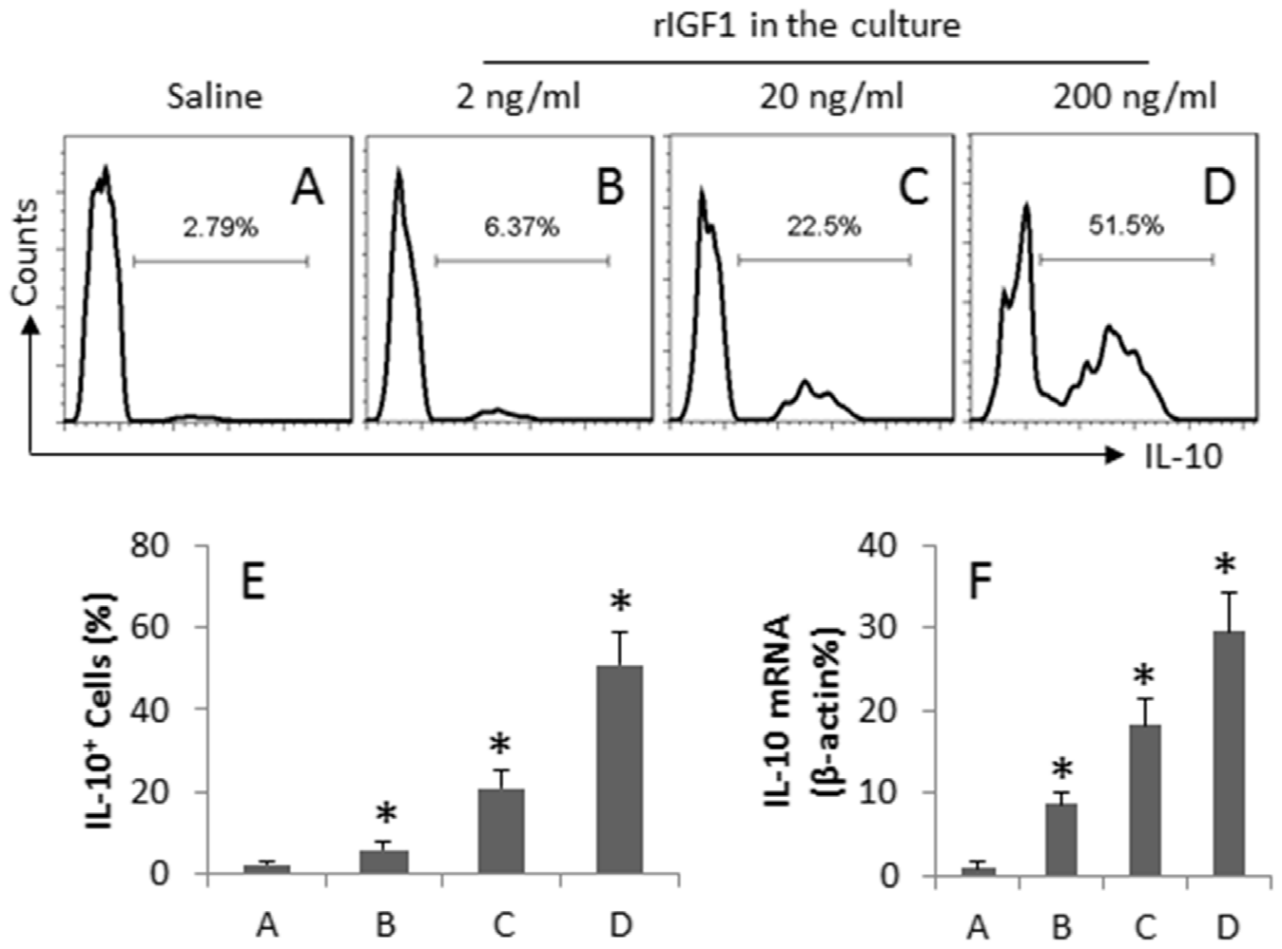
IGF1 induces IL-10^+^ Mos. CD14^+^ F4/80^-^ IGF1R^+^ Mos were isolated from the spleen. The cells were exposed to IGF1 (as denoted above each histogram) in the culture for 72 h and analyzed by flow cytometry and RT-qPCR. (A–D) the histograms indicate the frequency of IL-10^+^ Mos. (E) the bars indicate the summarized data of A–D. (F) the bars indicate the mRNA levels of IL-10. The data of bars are presented as mean ± SD. *, p < 0.01, compared with saline group. The data represent 3 independent experiments.

To further investigate the role of IGF1 in the regulation of the expression of IL-10 in Mos, the IGF1 receptor positive Mos were isolated from the spleen by MACS, and were exposed to IGF1 in the culture for 72 h. After exposure to IGF1 in the culture for 72 h, the frequency of IL-10^+^ Mos was markedly increased in an IGF1 dose-dependent manner (Fig. 3A–E). On the other hand, the RNA extracts of the Mos were prepared and analyzed by RT-qPCR. The results showed that the naïve Mos expressed lower, but detectable, levels of IL-10, which was markedly increased after exposure to IGF1 in the culture (Fig. 3F). The results demonstrate that IGF1 can enhance the expression of IL-10 in Mos.

**Figure 4:**
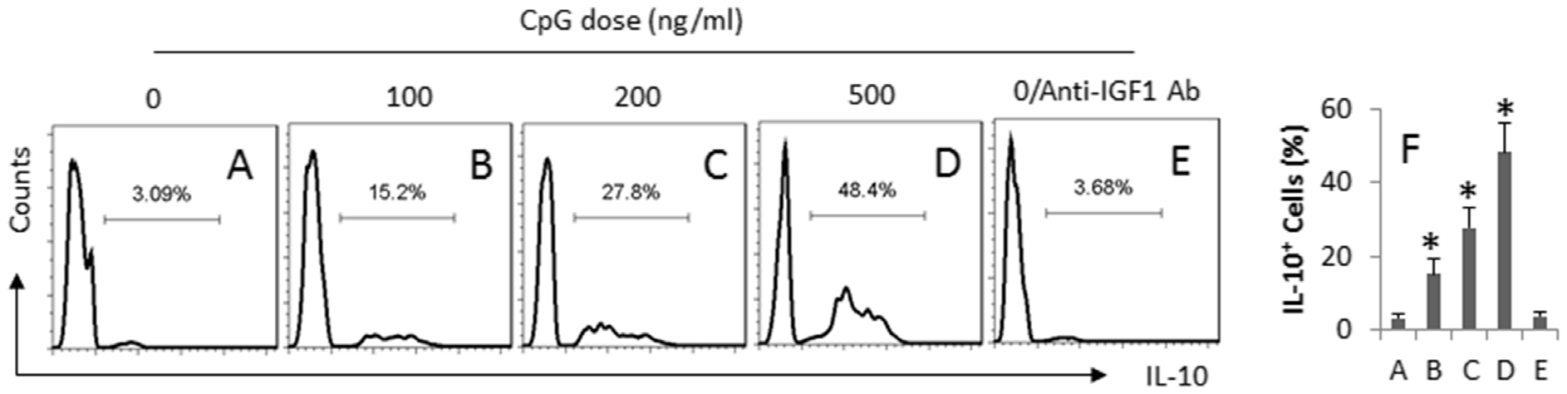
CpG induces IECs to produce IGF1 and induces IL-10^+^ Mos. Naïve Mos were cultured with IEC in a Transwell system (IECs in the inserts; Mos in the basal chambers) at a ratio of 1:1 in the presence of CpG (the doses of PMA are denoted above each histogram). The Mos were analyzed by flow cytometry. (A–E) the histograns indicate the frequency of IL-10^+^ Mos. (F) the bars indicate the summarized data of the IL-10^+^ Mos (mean ± SD. *, p < 0.01, compared with the dose ″0″ group). Anti-IGF1 Ab = 100 ng/ml. The data represent 3 independent experiments.

Alternatively, we cultured naïve Mos in the basal chambers of the Transwell system with IECs in the inserts. PMA was added to the apical chambers. The cells were cultured for 6 days. As shown by flow cytometry data (Fig. 4A–F), a low frequency of IL-10^+^ Mos was detected in the naive group (Fig. 4A), which was markedly enhanced after exposing to CpG-ODN in a CpG-ODN dose-dependent manner. The increase in IL-10^+^ Mos was blocked by the presence of anti-IGF1 antibody in the culture (Fig. 4E). The results implicate that the IEC-derived IGF1 can induce the expression of IL-10 in Mos.

### IGF1-primed Mos show immune suppressive effect

**Figure 5:**
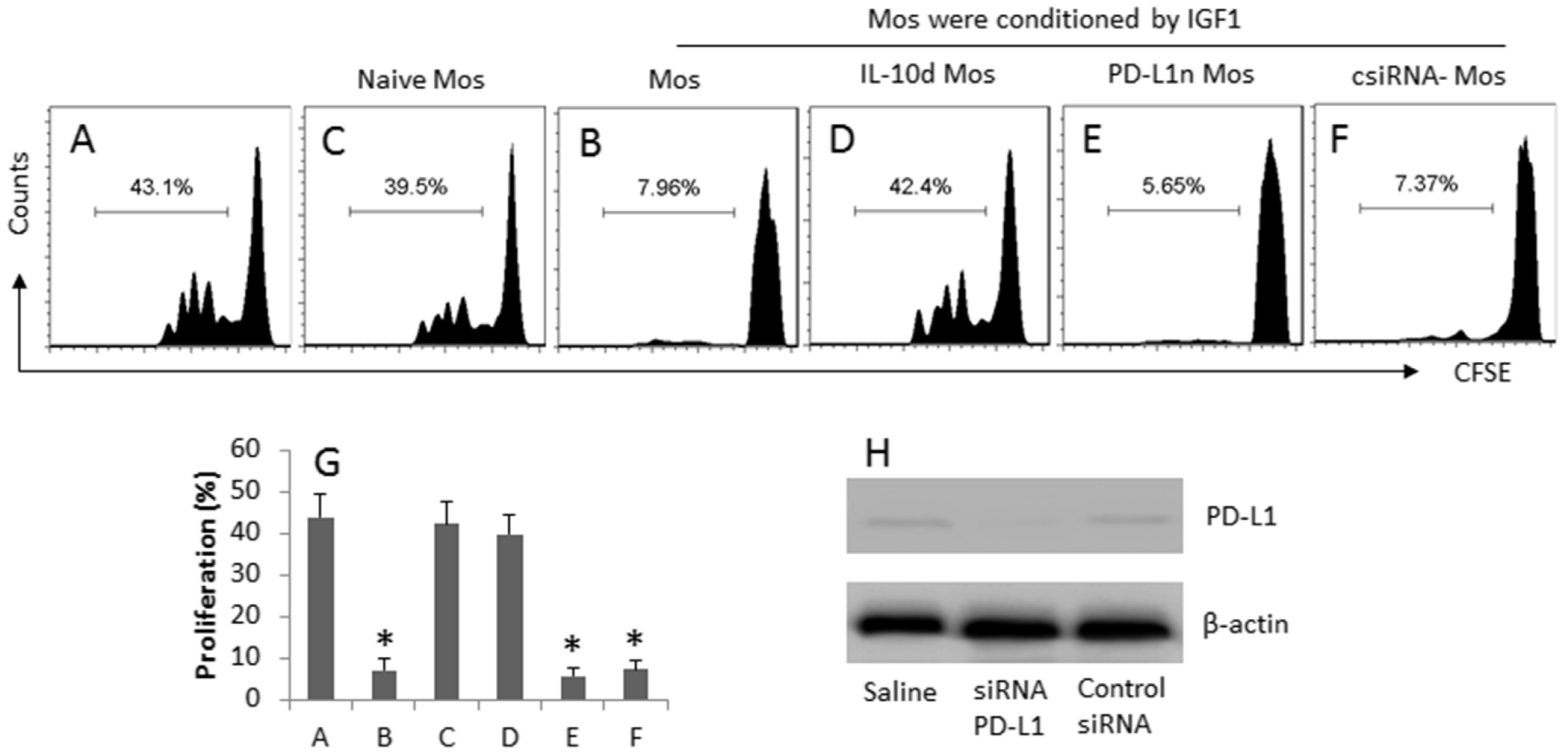
Immune suppressor functions of IGF1-primed Mos. (A) IGF1-conditioned Mos and Teff (CD4^+^ CD25^-^ T cells; labeled with CFSE) were cocultured at a ratio of 10^4^:10^4^/well in the presence of anti-CD3/CD28 for 6 days. The additional treatment was denoted above each histogram. (A–F) the histograms indicate the frequency of proliferative Teff. (G) the bars indicate the summarized data of A–F. (H) the Western blots indicate the results of PD-L1 RNAi of Mos. IL-10d: IL-10-deficient. PD-L1n: PD-L1-null. csiRNA-Mos: The Mos were treated with control siRNA. The data of G are presented as mean ± SD. *, p < 0.01, compared with group A. The data are a representative of 3 independent experiments.

Given the fact that the IGF1-primed Mos express high levels of IL-10 as shown by Fig. 4, the Mos may have similar immune suppressive functions of type 1 regulatory T cells. To test the inference, we cultured the IGF1-primed Mos and CD4^+^ effector T cells (Teff). As shown by flow cytometry data, exposure to anti-CD3/CD28 antibodies markedly induced the Teff proliferation (Fig. 5A, 5E), which was significantly inhibited by the presence of the IGF1-primed Mos (Fig. 5B, 5E), indicating the Mos suppressed the Teff proliferation. To strengthen the results, we cultured Teffs with naïve Mos with the same conditions of Fig. 5B; no apparent suppressive effect on the Teff proliferation was observed (Fig. 5C, 5E). To clarify the role of IL-10 in the immune suppressive functions of the Mos, an anti-IL-10 antibody was added to the culture of a separate experiment of the same condition of Fig. 5B. The results showed that the suppressive effect of the Mos was abolished (Fig. 5D, 5E).

### IGF1-primed Mos suppress colitis in mice

**Figure 6:**
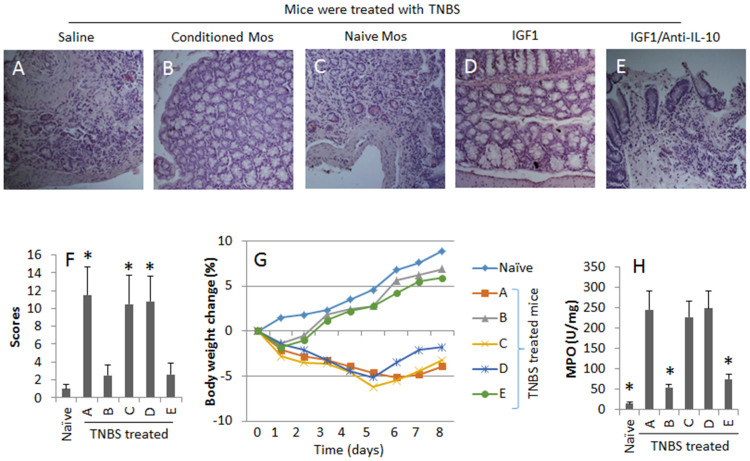
IGF1-primed Mos inhibit inflammation in the intestine. A TNBS colitis model was created with BALB/c mice. The IBD-associated parameters were evaluated with samples collected from the mice with the procedures described in the text. The additional treatments are denoted above each image. (A–E), representative colon histology images (Magnification: ×100). (F) the bars indicate the inflammatory scores. (G) the curves indicate the body weight changes. (H) the bars indicate the MPO levels in colon tissue. Mos-c: Mos were conditioned with IGF1. Mos-n: Naïve Mos. IGF1: Mice were injected (i.p.) with recombinant IGF1 (25 μg/mouse) on day 1 and day 3 respectively. The data of bars are presented as mean ± SD. *, p < 0.01, compared with the TNBS/saline group. Each group consists of 6 mice. Samples from individual mice were processed separately. The data are a representative of 6 independent experiments.

To test the suppressive function of the IL-10-expressing Mos, we created a colitis mouse model. The mice showed IBD-like inflammation in the colon (Fig. 6A–H). The adoptive transfer with IGF1-primed Mos, but not the naïve Mos, markedly improved the severity of intestinal inflammation, which was mimicked by administration with IGF1, and was abolished by administration with anti-IL-10 neutralizing antibody (Fig. 6A–H).

## Discussion

IBD is a greater negative impact on human health and social economy. Current therapeutic remedies are not satisfactory. Thus, to find novel remedies for the treatment of IBD is of significance. The present study shows a novel data set that has potential to make progress in the treatment of IBD. The data indicate that intestinal epithelial cells produce IGF1, the latter can be up regulated by exposure to CpG-ODN. The IEC-derived IGF1 induces the IL-10-expressing Mos; the latter inhibits the experimental colitis in mice.

The primary site of IGF1 production is the liver^13^. Growth hormone can increase the production of IGF1^13^. In turn, IGF1 can mediate the effect of growth hormone in body growth. IGF1 plays an important role in childhood growth and continues to have anabolic effects in adults. The present study has enriched our knowledge in the research of IGF1 by showing that mouse intestinal epithelial cells also can produce IGF1 and IGF1 can induce IL-10^+^ Mos.

Mos are a critical immune cell population in the body by showing its role in both innate immunity and adaptive immunity. The polarization of Mos is associated with the pathogenesis of a number of inflammatory disorders in the body; such as MIF (Mos migration inhibitory factor) is involved in the pathogenesis of IBD; administration with MIF blockade can attenuate the inflammatory process of IBD^24^. Recent studies indicate that Mos not only induces inflammation, an inflammation inhibitory subpopulation of Mos is identified^25^, in which Mos are such a subpopulation possessing anti-inflammation feature^25^. Thus, the generation of Mos in the body may be of therapeutic potential. Our data show that a fraction of the intestinal Mos expresses the IGF1R. The fact implicates that the intestinal Mos can respond to IGF1. The inference is supported by the subsequent data; by exposing to IGF1 in the culture, the Mos were differentiated to IL-10-producing Mos.

Another novel finding of this study is that the induced Mos to express IL-10. IL-10 is an immune regulatory molecule. Perez et al indicate that IL-10 also can induce the Tr1 regulatory T cell development^26^. In line with these pioneer works, our studies also demonstrate that IL-10-producing Mos can inhibit the experimental colitis. In order to test the functions of the IL-10-expressing Mos in vivo, we created a colitis model in the present study. The results showed that adoptive transfer with the IGF1-primed Mos inhibited the inflammation in the intestine. The data from this in vivo model further support the immune suppressive nature of the IGF1-primed Mos.

Our data indicate that exposure to CpG-ODN can increase the expression of IGF1 in IECs. Previous studies suggest that CpG-ODN can suppress experimental colitis. Hofmann et al indicate that CpG-ODN can relief experimental colitis via modulating dendritic cell functions^27^. Blaas et al suggest that CpG-ODN can induce intestinal B cell to secrete IgA indicating a link between adaptive and innate intestinal immune responses^28^. The present project provides additional information for the studies of CpG-ODN that CpG-ODN induces IECs to produce IGF1, the latter further contributes to the intestinal homeostasis by inducing Mos with immune suppressive properties. Our data are in line with the comments of Hofmann that the physiologic TLR9-CpG-ODN-DNA interaction is essential for the homeostasis of the intestinal immune system^20^.

In summary, the present study reveals that mouse intestinal epithelial cell-derived IGF1 can induce IL-10-expressing Mos in the intestine. The IL-10-expressing Mos show an immune suppressive function on experimental colitis in mice.

## Methods

### Reagents

Fluorescence-labeled antibodies of IL-10, CD14, CD80 were purchased from BD Bioscience (Shanghai, China). The shRNA of IGF1R reagent kit was purchased from abm (Richmont, BC, Canada). Recombinant IGF1 and anti-IGF1 antibodies were purchased from Santa Cruz Biotech (Shanghai, China). ELISA kits of IL-10 were purchased from R&D Systems (Shanghai, China). Reagents for quantitative RT-PCR and Western blotting were purchased from Invitrogen (Shanghai, China). Magnetic bead-conjugated streptavidin was purchased from Miltenyi Biotech (Shanghai, China). Transwells were purchased from Sigma Aldrich (Shanghai, China). The sequences of CpG-ODN-ODN and its control sequence were synthesized by GenScript (Shanghai, China). The sequence of CpG-ODN-ODN1668 is 5′-tccatgacgttcctgatgct-3′. The control sequence of GpG-ODN1668G is 5′-tccatgaggttcctgatgct-3′.

### Mice

Male BALB/c mice (8–12 week old) and IL-10-deficient mice were purchased from the Guangdong Experimental Animal Center. Mice were kept in a pathogen-free environment at Shenzhen University during the experimental period. The experimental procedures were approved by the Experimental Animal Ethic Committee at Shenzhen University; the methods were carried out in ″accordance″ with the approved guidelines.

### Intestinal epithelial cell culture

Mouse intestinal epithelial cell line, IEC4.1, was cultured in DMEM (Dulbecco's modification of Eagle's medium) supplemented with 10% fetal bovine serum, 2 mM L-glutamin, 100 U/ml penicillin and 100 mg/ml streptomycin. The medium was changed every 1–2 days. Transwell system was employed for a coculture experiment of IECs and Mos; IECs were seeded in the inserts while Mos were placed in the basal chambers.

### Quantitative real time RT-PCR (RT-qPCR)

Total RNA was extracted from IECs using the Trizol reagents following a protocol obtained from the manufacturer. cDNA was synthesized from 2 μg of total RNA using SuperScript III First-Strand Synthesis. PCR amplifications were performed on a MiniOpticon™ Real-Time PCR Detection System using SYBR® Green Master Mix. Fold change was calculated using the 2^−ΔΔCt^-method. The primers using in this study include: IGF1, forward, caactcccagctgtgcaatt; reverse, gccgaggtgaacacaaaact. IL-10, forward, ggtgagaagctgaagaccct; reverse, tgtctaggtcctggagtcca.

### Western blotting

Total proteins were extracted from the cells, fractioned in SDS-PAGE (sodium dodecyl sulfate polyacrylamide gel electrophoresis) and transferred onto a nitrocellulose membrane. After blocking with 5% skim milk for 30 min, the membrane was incubated with the primary antibodies (0.3–0.5 μg/ml) at room temperature for 1 h, and followed by the second antibodies (0.05–0.1 μg/ml; conjugated with horseradish peroxidase) at room temperature for 1 h. Washing with TBST (Tris-buffered saline with Tween-20) was performed after each antibody incubation. The immune complex on the membrane was developed with ECL (enhanced chemiluminescence). The results were photographed with a KODAK Image Station 4000 mm Pro (Shanghai, China).

### Isolation of immune cells from the intestinal segments

Lamina propria mononuclear cells (LPMC) were isolated as previously reported^29^. Briefly, intestinal segments were opened longitudinally and rinsed in PBS. Intestines were incubated with mild shaken in HBSS containing 5 mM EDTA and 5% fetal bovine serum (FBS) for 30 min at 37°C. After removal of epithelial layers, the intestines were cut into 2 × 2 × 2 mm pieces and incubated with RPMI 1640 containing 5% FBS, 1 mg/ml of collagenase D, 1 mg/ml of dispase and 40 μg/ml of DNase I for 1 h at 37°C with mild shaken. The digested tissues were washed with HBSS containing 5 mM EDTA. Cell suspensions were filtered through a 70 μm cell strainer; the cells were collected by centrifugation. The cells were re-suspended and were applied to gradient centrifugation at 1000 × g for 40 min. The cells were checked by trypan blue exclusion assay. The viability was greater than 95%.

### Flow cytometry

Cells were fixed with 2% paraformaldehyde (in the case of the intracellular staining, 0.5% saponin was added to the fixative to enhance the permeation of the cell membrane) for 30 min, washed with PBS for 3 times and incubated with the fluorescence-labeled antibodies (0.5–1 μg/ml) for 30 min at room temperature. After washing with PBS, the cells were analyzed by flow cytometry. 100,000 cells were counted for each sample. The data were analyzed with software Flowjo (Tree star, Ashland, OR).

### Isolation of immune cells

Immune cells were isolated by magnetic cell sorting (MACS) using commercial reagent kits following the manufacturer's instructions. The purity of the cells were greater than 95% as checked by flow cytometry.

### Induction of colitis and treatment of mice with Mos

Mice were treated by rectal injection of 2,4,6-trinitrobenzenesulfonic acid (TNBS) (2.5 mg/mouse) as previously described^30^.

### Histological scoring of colitis

The mice were sacrificed 4 weeks after the cell transfer. The transverse colons were removed from the mice and fixed with 4% paraformaldehyde, and embedded in paraffin. The tissue sections were stained with H&E stain or periodic acid–Schiff stain. The inflammation in tissue sections was scored following published criteria^31^. Histological inflammatory scores were recorded as follows: (A) the severity of inflammation: 0, none; 1, mild lymphoid infiltration; 2, marked lymphoid infiltration or focal degeneration of crypts; 3, severe inflammation or multifocal crypt degeneration and/or erosions. (B) The extent of inflammation: 0, none; 1, mucosal; 2, submucosal; 3, transmural; (C) amount of mucus: 0, normal; 1, slight decrease of mucus; 2, moderate decrease or focal absence of mucus; 3, severe depletion of mucus; 4, total absence of mucus; and (D) degree of cell proliferation: 0, none; 1, mild increase in cell numbers and crypt length; 2, moderate increase or focally marked increase; 3, marked increase in entire section. The inflammatory scores were the sum of the four individual parameters.

### Enzyme-linked immunobsorbent assay (ELISA)

The sera were collected from mice at sacrifice. The serum levels of IL-10 and IFN-γ were determined by ELISA with commercial reagent kits following the manufacturer's instruction.

### T cell proliferation assay

CD3^+^ CD4^+^ CD25^-^ T cells (Teff cells) were isolated from the mouse spleen and labeled with CFSE (carboxyfluorescein diacetatesuccinimidyl ester). The Teff cells were cultured in a plate coated with anti-CD3 antibody (2 μg/ml), anti-CD28 antibodies (5 g/ml) was added to the culture. Three days later, the cells were analyzed by flow cytometry.

### Myeloperoxidase activity

Myeloperoxidase (MPO) activity was measured with a myeloperoxidase assay kit according to the manufacturer's instructions.

### RNA interference

PD-L1 specific siRNA was designed and synthesized by Invitrogen (Shanghai, China) with the sequence of gggcgtttactatcacggctccaaa. The naïve Mos was transfected with the PD-L1 siRNA with lipofectamine (Invitrogen) following the manufacturer's instructions. The control Mos were transfected with a control siRNA that did not target on any genes. The gene knockdown effect was checked by Western blotting.

### Statistical analysis

Data are expressed as mean ± SD. Differences between two groups were determined by Student t test. In the case of more than two groups, one-way ANOVA was employed. When the results of ANOVA were significant, the Tukey–Kramer honestly significant difference test was applied for multiple comparisons. p < 0.05 was set as a criterion of significance.

## Author Contributions

R.T.G., L.H.M., R.W., J.Q.L. and H.P.Z. performed experiments, analyzed data and reviewed the manuscript. P.C.Y., Z.L. and Z.G.L. organized the project and supervised the experiment. P.C.Y. designed the project and wrote the manuscript.

## Supplementary Material

Supplementary Information supplemental materials
