# Supplementary material for: Insulin-like growth factor-1 endues monocytes with immune suppressive ability to inhibit inflammation in the intestine
Source: Sci Rep. 2015 Jan 15;5:7735. doi: 10.1038/srep07735 (PMC4295102; doi:10.1038/srep07735)
Supplement: Supplementary Information — supplemental materials [file srep07735-s1.pdf]

Supplemental materials

Insulin-like growth factor-1 endues monocytes with immune suppressive ability to inhibit inflammation in the intestine

Running title: IGF1 modulates monocyte property

Rong-Ti Ge*#, Li-Hua Mo¶, Ruijin Wu*, Jiang-Qi Liu#, Huan-Ping Zhang#, Zhigang Liu¶, Zhanju Liu*, Ping-Chang Yang¶#

*Department of Gastroenterology, The Shanghai Tenth People's Hospital of Tongji University, Shanghai, 200072, China. ¶Shenzhen Key Laboratory of Allergy & Immunology, Shenzhen University School of Medicine and State Key Laboratory of Respiratory Disease for Allergy at Shenzhen University, Shenzhen, 518060, China. #Brain Body Institute, McMaster University, Hamilton, ON, Canada L8N 4A6.

Ge RT, Wu R and Mo LH share the first authorship

Correspondence to: Dr. Ping-Chang Yang, Dr. Zhanju Liu and Dr. Zhigang Liu (301 Yanchangzhong Road, Shanghai 200072, China. 3688 Nanhai Ave, Shenzhen 518060, China. Email: liuzhanju88@126.com; pcy2356@163.com; lzg195910@126.com. Tel: +86 21 66301164; Fax: +86 21 66303983).

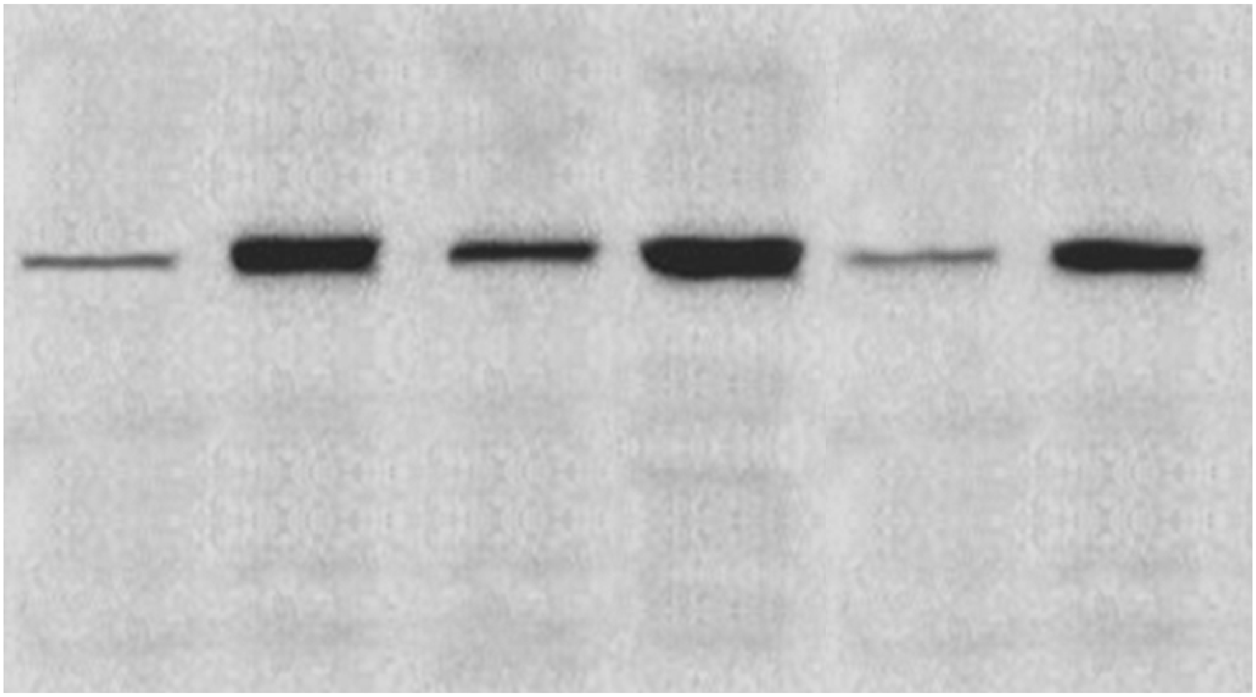

Figure 1B. IGF1 protein in IEC.

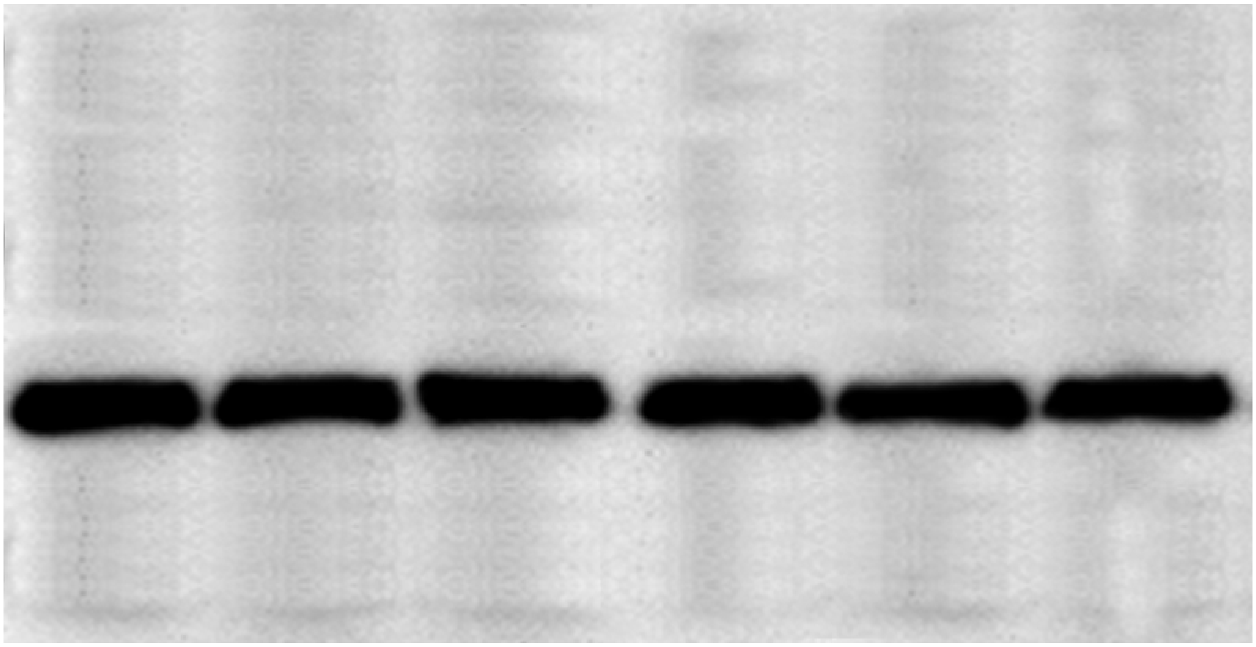

Figure 1B. Beta-actin.

Figure 5. PD-L1

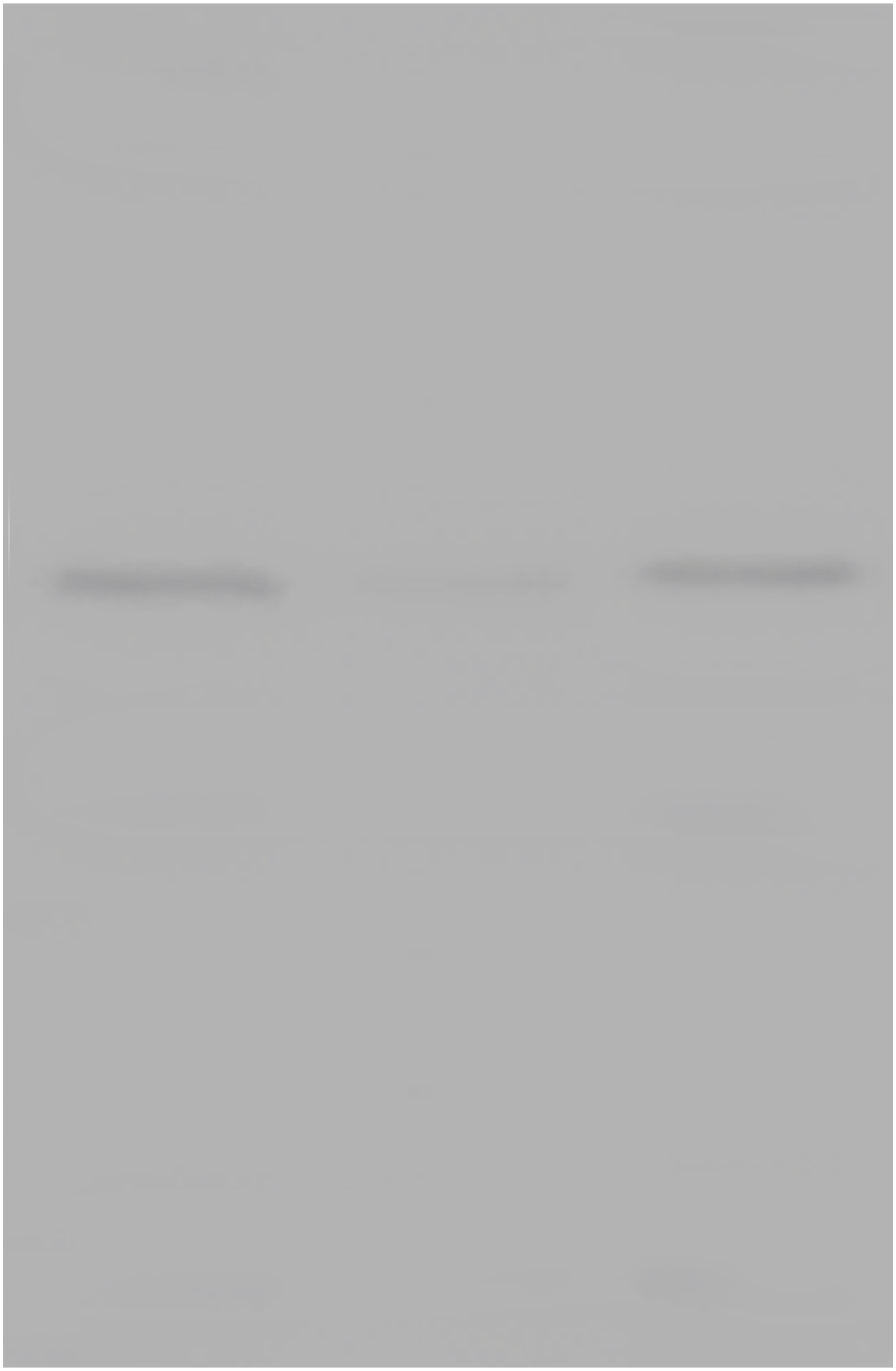

Figure 5. PD-L1

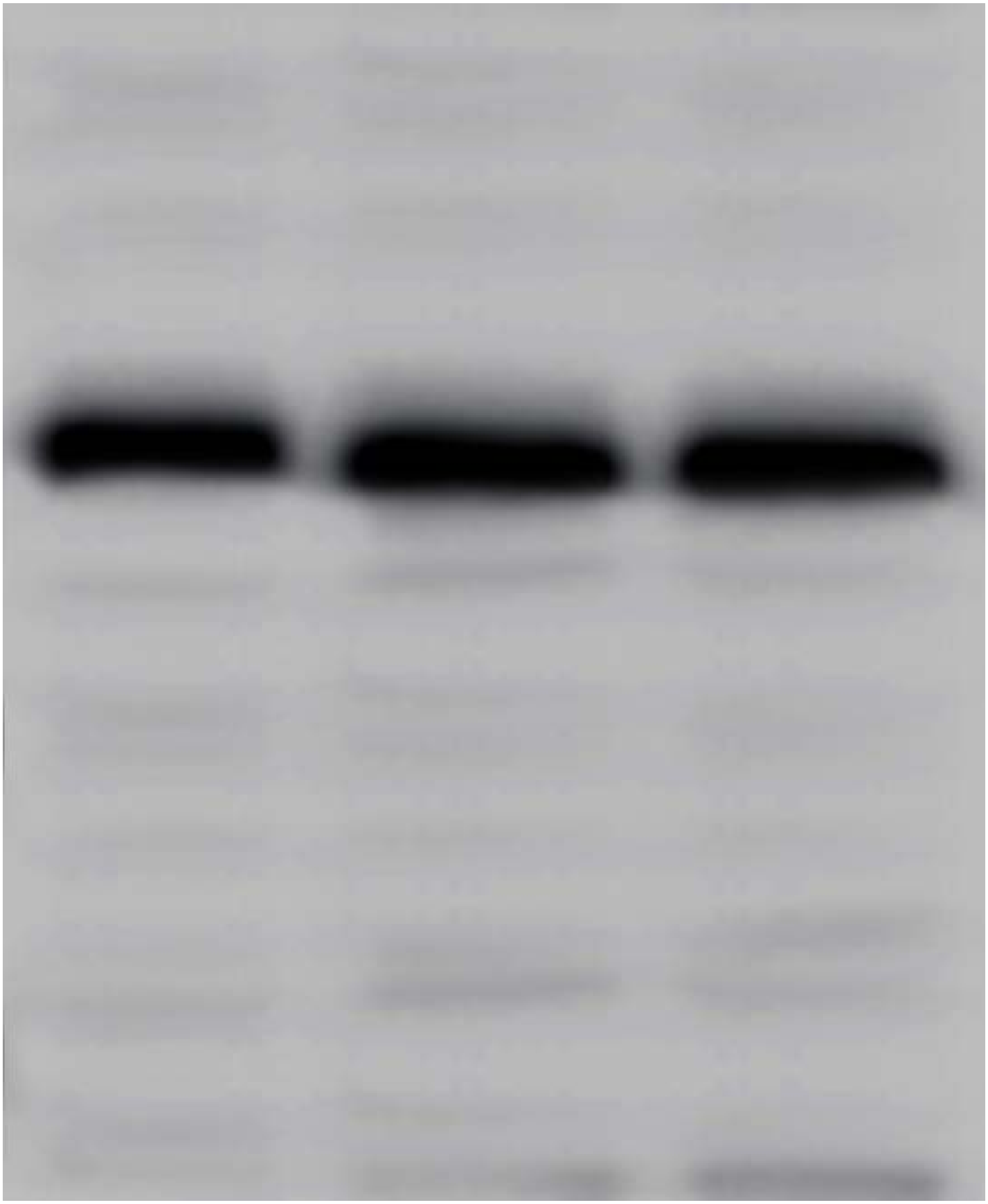

Figure 5. Beta actin
